# Becoming a coach: experiences of faculty educators learning to coach medical students

**DOI:** 10.1186/s12909-020-02119-z

**Published:** 2020-07-01

**Authors:** Joanna Veazey Brooks, Kathryn Istas, Bradley E. Barth

**Affiliations:** 1 Department of Population Health, University of Kansas School of Medicine, 3901 Rainbow Blvd, Mail Stop 3044, Kansas City, KS 66160 USA; 2 Information and Evaluation Resource Specialist, Office of Medical Education, University of Kansas School of Medicine, Kansas City, USA; 3 Department of Emergency Medicine, Assistant Dean of Student Affairs, University of Kansas School of Medicine, Kansas City, USA

**Keywords:** Medical student coaching, Curricular change, Faculty identity, faculty development

## Abstract

**Background:**

Despite the growth of coaching in medical education, many questions remain about the process of becoming a coach for medical students. We sought to understand the process through which faculty acclimated to this new role, and what benefits and challenges they experienced.

**Methods:**

A multi-phase qualitative focus group study was conducted with 20 faculty at one medical school in the United States during the initial year coaching was implemented. Focus group transcripts were analyzed using the constant comparative approach to inductively identify themes from the data.

**Results:**

Four main benefits were reported by faculty: student guidance, identifying student issues early, helping students develop work-life balance, and fostering clinician connectivity, which was seen as benefitting both students and faculty. The two main challenges were uncertainty regarding how adaptive the coaching sessions should be, and difficulty engaging in some of the roles simultaneously, like mentoring and supervision.

**Conclusions:**

Schools that develop academic medical student coaching programs should design faculty support around coaching and recognize that the process of becoming a coach may not be or feel straightforward for faculty. Overall, faculty found coaching to be rewarding despite challenges they experienced.

## Background

Coaching is a well-accepted method of developing leaders in the business community and is increasingly used in developing educators [1, 2]. In recent years, coaching learners has become more common in medical education curricula [3–9]. One-on-one coaching is proven to support medical learners' success academically and to facilitate individual self-knowledge, goal-setting, work-life balance, and reflection [3, 9, 10]. Coaching contributes to protection against burnout [1, 11–13] and fosters professionalism [4, 7, 11] by helping learners develop healthy habits and by addressing concerns early within the context of a trusted relationship. The American Medical Association (AMA) Coaching handbook has identified coaching as a “new and significant opportunity in medical education” [9].

While early reports show potential benefits of coaching, its adoption in undergraduate medical education (UME) is relatively recent. Review of the literature shows stronger evidence for coaching for technical skills compared to non-technical skills, and calls for further research into the “power” of coaching in medical education [3]. One central question around the implementation of coaching is how adept faculty are to changing from traditional teacher and mentor roles to one of coaching in which they iteratively collect information and provide feedback in an individualized manner [2, 3]. Most qualitative data thus far has reported on the student or coachee’s experience of coaching interventions [3, 6, 7, 14]. Only recently has attention begun to focus more on the experience of the faculty coaches. For example, one recent study considered the important role of social learning and community development among coaches as they learn from each other [15]. Our study continues to build on the base of knowledge about the faculty experience of coaching by investigating the process through which faculty learn how to be a coach, and the way that their interactions with students during coaching sessions informs that process.

We studied the experiences of faculty members in their first year of serving as an academic coach at a large, state medical school. We used focus groups conducted at different stages of the academic year to examine faculty members’ perceptions of the transition to academic coaching from previous roles. As more medical schools adopt coaching, our findings describe the benefits and challenges of the process of becoming a coach in order to inform best practices around coaching implementation.

## Methods

### Study setting

At the University of Kansas School of Medicine a new role of Learning Community Assistant Directors, colloquially referred to as “coaches,” was developed as part of a broader curriculum change to the Active Learning, Competency-Based, Excellence-Driven (ACE) curriculum in 2017 [16]. The purpose of coaching at our institution, as one component within ACE, was to provide increased exposure to physicians for students in the first 2 years and to provide protected space to address student questions and concerns that extended beyond traditional academic advising. Four coaches and one Director work together in each of the Learning Communities (LCs). The director serves as an assistant dean for student affairs, substitutes as a coach as needed, and provides oversight, advocacy, and accountability for coaches and students in their LC.

Faculty were informed about the opportunity to be a coach through a general announcement sent to all clinical faculty and specific invitations to apply. All coaches are active clinical faculty, and were chosen to reflect a diversity of specialties. Each coach is assigned six to eight first-year students, and coaches met one-on-one with their assigned students every 8-week block to develop and promote student success and wellness. By design, coaches have no role in assessment or decisions about progression for the students they coach. Faculty in this role received training through a number of sessions covering different topics, including resources for students (financial, counseling, student health, learning skills, etc.), rules and regulations for interacting with students (FERPA, LCME, etc.), small group facilitation, problem-based learning instruction, learning management software and documentation requirements, etc. Included in this training period was approximately 6–8 h of instruction and practice regarding coaching skills and methods. Coach training was based on understanding the difference between coaching and supervising, teaching, or mentoring behaviors and focused on open-ended questions and reflective listening. Coach specific training was led by an International Coaching Federation certified coach and followed an abridged version of their competency framework.

### Study design

We conducted a multi-phase focus group qualitative study of coaches. The focus groups were conducted on campus at three points during the first academic year of the program (Table 1). Two focus groups were conducted after 11 weeks of class; one at midyear; and two at the close of the year. The first two focus groups included both coaches and directors. In subsequent groups, coaches were separated from directors to avoid any power dynamics created by the reporting structure which could impact the discussion [17]. The script covered preparation for the role; the logistics of coaching (the tools, timing, and training); and the topics covered during coaching sessions while allowing for other topics to be generated by participants. The University of Kansas Medical Center Institutional Review Board approved the study.

**Table 1:** Respondents by Focus Group

Early in year	Mid way in year	Late in year
FG1-Coaches (*N* = 3)	FG3-Coaches *N* = 6	FG4-Coaches (*N* = 3)
FG2-Coaches (*N* = 8) Directors (*N* = 2)		FG5-Directors (*N* = 3)

### Recruitment and data collection

All academic coaches (*n* = 30) received e-mail invitations to attend focus groups. Participation was voluntary, and participants provided written informed consent when they first arrived at the focus group. Participants were informed that they could stop participation at any time and that their identities would be protected in reports of the study findings. Focus groups were conducted on campus in a central location, and lunch was provided to participants to eat during the focus groups. Interviews were audio-recorded and lasted 52–66 min. Two of the authors (JVB and KI), with training in qualitative methods and focus groups, co-facilitated each session. One author (KI) took extensive notes during the focus groups. The focus group guide was minimally adapted over time in response to the data and the time in the academic year, an established practice in grounded theory [18, 19]. Audio-recordings were professionally transcribed.

### Data analysis

Using an interpretivist paradigm, qualitative analysis of transcripts followed a constant comparative method consistent with a grounded theory approach [20]. Themes identified from the data informed subsequent focus group questions. Once all focus groups were complete, the three authors independently conducted a round of open coding of all faculty focus group scripts. They then met to discuss emergent themes until consensus was achieved on key themes, organized into benefits and challenges of coaching. The authors utilized techniques to ensure credibility of our findings, including the process of independent coding and peer debriefing [21].

## Results

Five focus groups had a total of 25 participants, with five faculty members participating in more than one group; no single participant attended all three time points. The 20 unique participants included eleven women, nine men, sixteen Academic Coaches, and four learning community directors (Table 1). Respondent quotes are identified by the time of year when the focus group was conducted (early, mid, end) and focus group number (1–5).

### Coaching benefits

Faculty participants reported four main benefits of coaching: (1) providing guidance; (2) identifying problematic student issues early; (3) developing student work-life balance, and (4) fostering clinician connectivity.

#### Providing guidance

Coaches described their role as “providing guidance” for students:

*I see myself as helping them to navigate through the process [...] They need to get perspective and I’m there to help them get perspective*” [Early in year, FG1].

Another coach framed guidance as helping students “navigate the system” [Mid-year, FG3].

#### Identifying issues early

Coaches described a unique component of their role as early identification of students at risk and to “catch students with problems earlier” [Mid-year, FG3]. One director described this capability:

*We have been able to identify students who are struggling very early, as early as the first missed lecture or the first missed PBL. We immediately know what’s going on. We can immediately have someone either call the student or meet with the student and ask what’s going on. And you also have a mechanism for regular follow-up and feedback.* [late in year, FG5]

Faculty shared their experiences with this function of coaching:

*So if I noticed something wasn’t quite right or there were some failures, or there were some things going on in their personal lives, [it’s] a first line of defense to kind of be able to guide them in the right direction* [Late in year, FG4]

*We had a student [...] who was very, very disengaged and almost disinterested, and with time and multiple discussions with her coach, and with other folks, I think she actually became one...of the most engaged students.* [Late in year, FG5]

#### Developing student work-life balance

Coaches perceived their role as providing a unique opportunity to guide students in professional development beyond academic success. They described a central function of coaching in helping students learn to develop their own healthy life habits that could sustain them throughout their medical training and careers:

*I think helping to ground them, like to help them think of a good balance between … work-life balance, school work and life reminding them that hey, ‘let’s look at your goals other than study habits’.* [Late in year, FG4]

*I also would ask them not just about their classes, but are you eating healthy, are you exercising, are you keeping a balance? So, it was really to just to keep them moving forward; so I felt like it was to make sure that they were progressing well scholastically, but also to touch on the other things*. [Late in year, FG4]

Coaches described general conversations about work-life balance as well as helping students through specific challenges, including personal illness and death of a family member. One coach reported her conversation with a student who was a new parent:

*We had to have a conversation about making sure his wife, who has a new baby and stays at home all day in an apartment, gets some time to go to Target or something by herself. It's a culture shock for her to have this change where now her husband is gone and she has a baby. He does great on exams and he's got everything else together but that has been the one thing we've consistently worked on.* [Early in year, FG2]

For some coaches, understanding the broader aspect of coaching increased as the year progressed:

*It is about academics, but it’s also about developing them as whole people. I kind of thought the coaching was going to be about “go, study hard”, and then realizing that there are so many more nuances, and it really is much more individualized to their goals and who they want to be as physicians and then trying to develop that, [it] is way more interesting than just telling them to study*. [Late in year, FG4]

#### Clinician connectivity

Coaches believed this exposure to clinical faculty member coaches, “right off the bat” [Early in year, FG1] was helpful in professional socialization and that meeting with the same clinician-coach over time would benefit students.

*I thought it was a really nice way for each student to have sort of a personal touchstone; that they had somebody who they were accountable to, but who was also accountable to them* [Late in year, FG4]

Some faculty members contrasted the current coaching experience with their own experiences as medical students when they were more isolated from clinical faculty, especially in the first 2 years.

*Well I think, you know, they want to aspire to be like us, right? And if they don't have a role model, if they don't get to see other physicians who have already gone through what they're going through, that kind of gets lost in the shuffle of what they're aiming for. And so just to put a face to this is what I want to do. I want to be a practicing physician. I think it's important for them early on. As a medical student, I didn't get to know clinical faculty really much at all.* [Early in year, FG1]

Coaches also reported that one of the benefits of this connectivity between clinicians and students was that they were able to normalize the often anxiety-producing experience of medical school:

*I think with my group it’s more reassurance than anything. “This is normal. This is going to be OK. This is normal*.” (Early in Year, FG2)

*I think it also offers them some perspective. We've all been through med school and we can offer support. It's very trying and there are times when you're not going to do as well as you think you're going to do or you've done in the past because not everyone is the top 10%. Having to deal with failures as well as successes, we've all been through that so I think that offers some perspective for them.* [Early in year, FG1]

Finally, coaches reported personal benefits from connectivity as they built relationships with students.

*I think what’s happening as a result of us doing all of this is that we’re getting more—I feel like I’ve got more connection to this class than I have ever had to any medical school class in the past.* (Mid-Year, FG3)

*Especially, I mean, as an introvert I want to be able to make connections one-on-one, not in a group of 50 or 100, so it made me feel much more connected to the students and to the new curriculum.* [Late in year, FG4]

### Coaching challenges

Faculty described two main challenges: uncertainty about the structure of coaching sessions, and difficulty balancing some of the roles expected of the coach.

#### Uncertainty about structure of coaching sessions

Respondents reported feeling uncertain about flexibility of structure in coaching sessions. For example, coaches were instructed to have students create personal goals, but were met with variable amounts of success. The extent of flexibility in conducting coaching sessions also emerged in discussions of how to adapt coaching to individual students. Participants noted that they felt the need to tailor coaching sessions to individual students and to adapt approaches as the year progressed.

*At the beginning, when we had a little more structure on the coaching [...], it’s a level playing field to start with. So, it was a quite a lot of anxiety and we filled up the time, I think, almost every time for those first few sessions. Then as they start, like in a race, spreading out, and some of them are doing more poorly and some better, that amount of time changed. [ … ] It really varies according to the person.* [Mid-year, FG3]

The focus group of directors at the end of the year discussed the tension between structure across coaching while maintaining responsiveness to individual student needs:

*I do think it would be beneficial for us to build some more topics, goal topics to hit [...] for this block we talk about this, this block we talk about this. I do think that would be helpful for people. Again, I do hesitate to push things on the [coaches]. To allow for that autonomy, to allow for the things that they feel are necessary to take place, [ … ] they don’t have to talk about things that they don’t find helpful.* [Late in year, FG5]

The respondents in this group went on to say that they did not want the suggestions to be “so structured” or feel like a “script” but that “giving general topics might be helpful.”

#### Difficulty balancing roles

Coaches described tension in balancing the supervisory and mentoring roles:

*Finding that balance between being friend and being coach and mentor and supervisor is a little challenging. We are there to coach them up, to pick them up when things are going bad or good for that matter, but also to serve as supervisory role if they’re not*

*attending classes or if there are things that they're required they're not doing. [ … ]that can be a little tricky navigating, to be someone that they can confide in but also someone that does have authority.* [Early in year, FG1]

*I think for a coach to be effective, I think the coach needs to be friendly and accessible to the student on the one hand, but also needs to be somewhat detached and objective. If you are not friendly and accessible, you will not have a good interaction and I don’t think the student will have a positive interaction, and may not necessarily open up, especially when there are sensitive things going on—and they wouldn’t trust you. But also, if you’re not somewhat detached and objective, then you end up with that slippery slope where it becomes a personal relationship, and I don’t think the role is designed to be a personal relationship...* [Late in year, FG5]

While most coaches agreed that this balance was needed, it was not always easy to navigate.

## Discussion

We examined the experiences of faculty during their first year as coaches through focus groups. Respondents identified four key benefits: providing guidance, identifying student issues early, developing and maintaining balance for students, and clinician connectivity. Coaches also reported two challenges: uncertainty about the ideal structure for coaching sessions, and difficulty balancing multiple roles.

Faculty in our study did express some uncertainty about their new role, but this uncertainty resolved for the most part as the year progressed. As would be expected, coaches were more likely to reflect on their own benefit of connectivity to the class in the later focus groups, after they had time to build relationships with students. With increased experience, each coach began to identify her own personal approach to the role and reported feeling more comfortable with overall experience. The tension about the degree of structure for sessions seemed to increase as the year progressed, possibly because the range of student needs expanded, for example high-achieving students needed different support than those students who were struggling personally and/or academically. Apart from these shifts over time, the themes coaches reported were remarkably consistent throughout the year.

Several of the benefits or functions of coaching found in our study are consistent with previous research [5, 7]. While feeling more connected to students may improve faculty satisfaction and wellbeing, it is too soon to tell the impact of coaching on lifelong skills and levels of burnout [1, 22]. While not the purpose of the our research focus groups, participants seemed to use the forum as a type of peer debriefing, gathering ideas and suggestions from other coaches, a “social learning” that other coaching programs have shown to be valuable [15]. Schools implementing coaching may want to consider designing modalities for coaches to discuss challenges in a supportive environment.

Our study has limitations. This was a single institution study, and coaching programs that currently exist are variable across institutions, which limits the generalizability of our findings, especially for coaching programs that differ substantially in their goals or structure of coaching programs. While our sample is too small to be able to draw meaningful conclusions about coaching experiences based on specific demographic variables (age, gender, race, specialty), future research should consider how these identities, and their intersectionality, may shape coach experiences. Because the new coach position was part of a major change in approach to education, it is difficult to separate faculty’s experiences of transition to the coach role from transition of the overall environment of curriculum change. Additionally, we cannot be certain that the faculty participants represent the experiences and perspectives of faculty coaches who did not participate.

## Conclusions

While coaching is still relatively uncommon in UME, it is associated with improved student experience and enhanced professionalism and socialization as well as benefits to faculty participants. Our research highlights the value of coaching for the involved faculty, who reported increased connectivity to students, as well as the benefit to student affairs by increasing awareness of potential student concerns. Our findings suggest several best practices for schools implementing coaching programs. First, training for coaches should make clear the differences between traditional faculty roles, like teaching or mentoring, and coaching. Faculty development should target specific coaching skills, such as active listening and asking powerful questions, that may feel particularly difficult for new coaches. Ultimately, faculty training for coaching should prepare faculty to think and act like a coach, instructing them on how to *become* a coach, and not assuming that faculty will find coaching easy or natural at first [22]. Sharing first-hand examples from colleagues of their benefits and challenges may be particularly helpful. Finally, our study suggests the importance of providing space for periodic feedback—especially after coaching sessions with students have begun, which will provide an opportunity to tailor training to any specific issues that arise, and to foster peer learning.

## Data Availability

The qualitative data collected by focus groups does not permit the sharing of audio recordings due to respondent confidentiality concerns. If there are specific questions about the data, authors can potentially provide additional de-identified responses.

## Abbreviations

AMA: American Medical Association
UME: Undergraduate Medical Education
ACE: Active Learning, Competency-Based, Excellence-Driven
LC: Learning Communities
PBL: Problem Based Learning
